# Effect of optimized germination technology on polyphenol content and hypoglycemic activity of mung bean

**DOI:** 10.3389/fnut.2023.1138739

**Published:** 2023-04-03

**Authors:** Bo Li, Xinting Shen, Huifang Shen, Ye Zhou, Xinmiao Yao

**Affiliations:** ^1^Food Processing Research Institute, Heilongjiang Academy of Agricultural Sciences, Harbin, China; ^2^Heilongjiang Province Key Laboratory of Food Processing, Harbin, China; ^3^Heilongjiang Province Engineering Research Center of Whole Grain Nutritious Food, Harbin, China

**Keywords:** mung bean, sprouting, polyphenol, hypoglycemic, T2DM

## Abstract

The study aimed to investigate the effect of germination conditions on the content of polyphenol extract in mung bean and to further investigate the effect of polyphenol extract in germinated mung bean on diabetic mice. Through single factor experiment and response surface experiment, the effects of soaking temperature, soaking time, germination temperature, germination time and soaking liquid CaCl_2_ concentration on the polyphenol content of mung bean were analyzed. The optimal germination conditions of mung bean were determined as soaking temperature 25°C, soaking time 11 h, germination temperature 28°C, germination time 3 days and CaCl_2_ concentration 2 mM. Under these conditions, the content of polyphenol extract in germinated mung bean was 4.878 ± 0.30 mg/g, which was 3.07 times higher than that in ungerminated mung bean. The structure and content of purified polyphenols in germinated mung bean were determined by HPLC-MS/MS. Quinic acid, Quercetin, Rutin, Vitexin, Isovitexin and other substances were identified, and the content of polyphenols was 65.19%. In addition, through the *in vivo* and *in vitro* hypoglycemic activity experimental study of germinated mung bean polyphenols extract, the results showed that germinated mung bean polyphenols had an *in vitro* inhibitory effect on α-glucosidase, IC_50_ was 44.45 mg/ml. *In vitro* inhibitory activity was stronger after digestion. Polyphenol extract can significantly reduce blood sugar and improve insulin resistance in Type 2 diabetic mice (T2DM). According to the results, germination treatment is an effective way to increase the content of polyphenols in mung bean, and the polyphenols extract has hypoglycemic activity.

## 1. Introduction

Mung bean is one of the most important edible legumes (1), and it has high nutritional value (2). In addition to containing important staple nutrients, such as proteins and carbohydrates, mung beans, contain bioactive components, such as polyphenols, coumarins, alkaloids, saponins, and phytosterols, which perform pharmacological function (3–5). Studies have confirmed the health benefits of various chemical components of mung bean, particularly polyphenols, polysaccharides, and peptides (6–8). Diabetes is caused by the disorder of human glucose metabolism, which makes the body in a state in which glucose metabolism and lipid metabolism are improperly regulated by insulin, which can lead to the increase of fasting and postprandial blood glucose. Type 2 diabetes is more common, accounting for 75% ~ 85% of all diabetes cases. But at present, most of the drugs to treat diabetes have some side effects, so the development of a natural drug with less side effects has become the focus of diabetes research. In recent years, studies have found that polyphenols have a good hypoglycemic effect, and polyphenols are the most common components of plants (9). Studies have shown that legumes are an important source of phenols. At present, there are few studies on the content and functional activities of polyphenols in mung beans before and after germination, and the development of natural active substances for lowering blood sugar has far-reaching significance for the prevention and treatment of various diseases (8, 10). As one of the processing methods of coarse grains, germination treatment can significantly improve the nutritional value and the content of bioactive components and active metabolites in mung bean (1). During the germination of the mung bean, many substances are degraded and converted into substances that are more easily absorbed by the human body. Germinated mung beans therefore contain more functional substances than non-germinated mung beans (11). After germination, the total contents of phenolic acids and flavonoids (including vitexin and isovitexin)were significantly increased to 4.5-and 6.8-times that of raw mung bean seeds (12, 13). Moreover, the antioxidant activity and hypoglycemic activity of mung beans were also improved after germination. Therefore, this study has practical implications for enriching functional products of beans.

In this experimental study, we aimed to investigate the optimal conditions for increasing the polyphenol content in mung beans through germination treatment. We explored the effects of soaking temperature, soaking time, germination temperature, germination time and the concentration of CaCl_2_ in the soaking solution on the polyphenol content in mung beans. The hypoglycemic activity of germinated mung bean polyphenol extract was evaluated through *in vivo* and *in vitro* experiments. Then, the effects of germinated mung bean polyphenol extract on hypoglycemic activity of diabetic mice were demonstrated by comparing the blood glucose, glucose tolerance, insulin level and pathological sections of liver tissues of diabetic mice after different doses of instomach administration. This study provided theoretical support for further research on the development of safe and efficient hypoglycemic functional products using germinated mung bean as raw material.

## 2. Materials and methods

### 2.1. Materials and chemicals

Mung beans were purchased from Daqing Ruifeng Agricultural Technology Co., Ltd (Daqing, China). Absolute ethanol was purchased from Liaoning Quanrui Reagent Co., Ltd. (Liaoning, China). Gallic acid and anhydrous calcium chloride were purchased from Tianjin Fuchen chemical reagent factory. VC standard and Fulin phenol was purchased from Shanghai Yuanye Biotechnology Co., Ltd (Shanghai, China). All other reagents were analytical grade. STZ was purchased from sigma of the United States; Metformin hydrochloride was purchased from Shanghai Yuanye Biotechnology Co., Ltd (purity>99.9%), and basic feed and high-fat feed were purchased from TROPHIC Animal Feed High-Tech Co. Ltd, China. Feed code: TP23300, C57BL/6 mice (4 weeks old), with an average weight of (18 ~ 20) g, provided by Liaoning Changsheng Biotechnology Co., Ltd (Liaoning, China). The animal use license number is scxk (Liaoning) 2020–0001.

### 2.2. Mung bean germination procedure

Fresh mung beans are selected and rinsed with water. Mung beans were first soaked in warm water at 50°C for 1 min, and then rinsed with cold water, which was beneficial to activate dormant mung bean seeds and facilitate germination of mung beans. Then spread the soaked mung beans evenly on the petri dish, moisten the gauze and place them on the mung beans, and put them in the incubator for germination treatment. During the germination process of mung bean, some samples were taken out and cleaned with distilled water, drained and placed in a drying oven at 40°C for drying. After drying, the powder was beaten and screened to obtain germinated mung bean powder. The polyphenol content of mung bean in the germination process at different times was measured. Three parallel measurements were made each time.

### 2.3. Extraction and content determination of polyphenols

Polyphenols were extracted from the germinated mung beans, and the germinated mung bean flour was weighed and placed in a beaker. According to the solid–liquid ratio of 1:26 (g/mL), 70% ethanol solution was added and fully mixed at room temperature for ultrasonic treatment. The conditions were set as ultrasonic power 350 W, ultrasonic temperature 30°C and ultrasonic time 30 min. The solution was centrifuged immediately after ultrasonic treatment, and the centrifugal speed was 5000 r/min for 15 min. After centrifugation, the supernatant in the centrifuge tube was collected, and the steps of collecting supernatant after centrifugation were repeated for three times. The supernatant obtained after centrifugation was combined to obtain the crude polyphenol extract to be tested.

The polyphenol content was determined according to the Folin–Ciocalteu method with some modifications (14, 15). Specifically, 1 ml of polyphenol extract from germinated mung bean was placed into a 10 ml volumetric flask and 0.5 ml Folin-Clocalteu phenol reagent was added. Within 4-6 min, 1 ml of 7% Na_2_CO_3_ solution was added, followed by shaking and addition of distilled water up to 10 ml total volume. The sample was incubated in the dark for 1 h, and the absorbance value of the sample at 760 nm was determined. The mass concentration of polyphenols in the solution was calculated using the standard curve, with 3 parallel samples prepared for each group. A gallic acid standard curve was prepared according to the experimental method of Ahmed Hiwa M (16). The linear regression equation was y = 0.1075x + 0.0049 (R^2^ = 0.9999). The calculation formula for polyphenol content of samples (mg/g) = (C × V × N)/M, where C represents the mass concentration of germinated mung bean polyphenols (mg/mL), V represents the volume of sample extract (mL), N represents dilution multiple, and M represents the sample mass (g).

### 2.4. Single-factor and response surface optimization experiment

The effects of soaking temperature, soaking time, germination temperature, germination time and CaCl_2_ concentration of soaking solution on polyphenol content of germinated mung beans were investigated by single factor experiment. The effects of soaking temperature 15, 20, 25, 30, and 35°C on the contents of polyphenols in germinated mung bean were investigated under the conditions of soaking time 12 h, germination temperature 25°C, germination time 3d and soaking solution CaCl_2_ concentration 2 mmol/L, respectively. The effects of soaking time for 4, 8, 12, 16 and 20 h on the content of polyphenols in germinated mung bean were investigated under the conditions of soaking temperature 25°C, germination temperature 25°C, germination time 3d and soaking solution CaCl_2_ concentration 2 mmol/L. The effects of 15, 20, 25, 30 and 35°C on the content of polyphenols in germinated mung beans were investigated under the conditions of soaking at 25°C, soaking for 12 h, germinating for 3d and soaking solution CaCl_2_ at 2 mmol/L. Under the conditions of soaking temperature 25°C, soaking time 12 h, germinating temperature 25°C and soaking solution CaCl_2_ concentration 2 mmol/L, the effects of germination time 1, 2, 3, 4 and 5 days on the content of polyphenols in germinated mung bean were investigated. Under the conditions of soaking temperature 25°C, soaking time 12 h, germinating temperature 25°C and germinating time 3d, the effects of soaking solution concentration 0, 1, 2, 3, and 4 mmol/L of CaCl_2_ on the content of polyphenols in germinated mung bean were investigated. The content of polyphenols in dried sprouted mung bean powder was determined according to the extraction method of polyphenols. The experiment was conducted three times in parallel.

According to the results of single factor test, soaking temperature, soaking time, germination temperature, germination time and CaCl_2_ concentration were further optimized, and polyphenol content was used as the index for comparison. The design expert 8.0.6 software was used to conduct regression analysis on the experimental data, determine the quadratic linear regression equation, and determine the optimal process conditions. The experimental scheme designed by response surface optimization analysis is shown in Table 1. The experimental results were analyzed by analysis of variance and response surface, and the response surface model was used to determine the best conditions for mung bean germination.

**Table 1:** Response surface test factors and levels.

Level	Factor				
	Soaking temperature (X_1_)/°C	Soaking time (X_2_)/min	Germination temperature (X_3_)/°C	Germination time (X_4_)/d	CaCl_2_ concentration (X_5_)/mmol/L
−1	20	8	25	2	1
0	25	12	30	3	2
1	30	16	35	4	3

### 2.5. Separation and purification of polyphenols

The referred method was utilized with some modifications (17, 18). The AB-8 macroporous adsorption resin was sealed and soaked in 95% ethanol for 24 h, then loaded into the column, washed with deionized water to completely eliminate the ethanol, then washed with 5% HCl solution and 5% NaOH solution for 3 Bv and soaked for 4 h. Finally, the macroporous resin was washed with deionized water for further experiments. Macroporous resin (5 g) and germinated mung bean polyphenol solution (50 mL) were placed into the triangular grinding flask, and shaken on a shaking table set at 25°C, and 160 r/min. During the experiment, samples were taken at 1 h intervals. A sample volume of 1 ml supernatant was used to determine the concentration of polyphenols. The calculation formula for static adsorption capacity was as follows:Static adsorption capacity (mg/g)= $\frac{\left(C_{0}-C_{1}\right)\times V_{1}}{M}$ _._ C_0_ is the initial mass concentration of the crude extract of germinated mung bean polyphenols (mg/mL), C_1_ is the mass concentration of polyphenols in the solution after adsorption equilibrium (mg/mL), V_1_ is the initial volume of polyphenol crude extract of germinated mung bean (mL), and M is the mass of macroporous resin (g).

The surface solution of the saturated macroporous resin was washed with deionized water, blotted with filter paper, placed into a triangular flask, and 50 ml of absolute ethanol solution was added. The solution was placed on a shaker, and samples were taken every 1 h. A sample volume of 1 ml supernatant was used to, determine the concentration of polyphenols. The calculation formula for static desorption rate was as folllows: Static desorption rate (%)= $\frac{C_{2}V_{2}}{\mathrm{MQ}}\times 100$ _._ C_2_ is the mass concentration of polyphenols in germinated mung bean (mg/ml), V_2_ is the volume of desorbed solution added (mL), M is the mass of macroporous resin (g), and Q is the adsorption amount (mg/g).

Different concentrations of germinated mung bean polyphenols were selected for dynamic adsorption under the purification condition of 1.0 Bv/h. After the adsorption of microporous resin was saturated and balanced, the impurities in the column were washed with deionized water, and then the eluent was dynamically eluted at a flow rate of 2 Bv/h. The polyphenol adsorption quantity of the different solutions was analyzed to determine the most appropriate polyphenol concentration. Different volume fractions of ethanol solution were selected for elution. The effect of ethanol volume fraction on the desorption of macroporous resin was investigated to determine the most appropriate concentration of eluent. The purified germinated mung bean polyphenol solution was collected and concentrated in a 40°C rotary evaporator. The purified concentrated solution was pre-frozen in a 20°C refrigerator and placed in a vacuum freeze-drying machine, and lyophilized into powder for further experiments.

### 2.6. Structural analysis of polyphenols from germinated mung bean

The samples were dissolved in deionized water and filtered by 0.22 μm organic membrane. HPLC conditions: Chromatographic column: C18 column (250 mm × 4.6 mm, 5 μm) with octadecyl bonded silica gel as filler; Mobile phase A: acetonitrile; Mobile phase B: 0.1% formic acid aqueous solution; Flow rate: 1 mL/min; Column temperature: 25°C; Sample size is 10 μL. Detection wavelength: 280 nm. Mass spectrum conditions: dry gas pressure 50 psi; Temperature is 550°C; Declustering potential is 90 V; Collision energy is 10 V.

### 2.7. *In vitro* simulated digestion of polyphenol extracts from germinated mung bean

The experiment included two parts: simulated gastric juice digestion and simulated intestinal juice digestion.100 ml of the sample solution was used to adjust the pH to 2.0 with 1 mol/L HCl, and then 18 ml pepsin solution (500 U/mg) was added. The conical bottle was wrapped with aluminum foil and placed in a thermostatic shaking box, which was kept away from light. The digestion of gastric juice *in vitro* was simulated for 2 h at 37°C and 100 r/min. After the simulated gastric juice digestion reaction was completed, 50 ml of gastric juice digestion sample solution was taken, the pH was adjusted to 7.0 with 1 mol/L NaHCO_3_ solution, 18 ml trypsin solution (200 U/mg) was added, and the *in vitro* digestion was simulated for 2 h at 37°C and 100 r/min. The samples were heated in boiling water bath at 100°C for 5 min to terminate the enzymolysis reaction. The supernatant was centrifuged at 4°C and 12,000 r/min for 10 min, and the polyphenol content and hypoglycemic activity were determined.

### 2.8. Inhibition of α-glucosidase by germinated mung bean polyphenols *in vitro*

The experimental method was based on the protocol of others with modifications (19, 20). The lyophilized germinated mung bean polyphenols were prepared into solutions with different mass concentrations. The sample solution (10 μL) was mixed with α-glucosidase phosphate solution (45 μL, 5 U/mL), and then put into an incubator at 37°C for 10 min. Next, 35 μL of 5 mM 4-nitrobenzene-α-D-glucopyranoside solution was added, and the reaction was incubated at 37°C for 30 min. Na_2_CO_3_ solution (100 μL) was added to terminate the reaction. The absorbance of the solution at 405 nm was measured with an ultraviolet spectrophotometer. PBS solution was used to replace the α-glucosidase phosphate solution or the enzyme solution in the blank controls. In the calculation formula, A_C_ is the absorbance measured without adding the sample reaction solution; A_0_ is the absorbance value measured by blank enzyme solution; A_s_ is the absorbance of the reaction solution to which the sample is added; and A_b_ is the absorbance value of the blank sample. The calculation formula of α-glucose inhibition rate is as follows:


$$\alpha -\mathrm{glucose inhibition rate}\left(\%\right)=\frac{\left(A_{c}-A_{0}\right)-\left(A_{s}-A_{b}\right)}{\left(A_{c}-A_{0}\right)}\times 100$$

### 2.9. Hypoglycemic experiments on animals

#### 2.9.1. Establishment of a model of type 2 diabetes mellitus

Type 2 diabetic mice were established by high fat diet combined with intraperitoneal injection of STZ (30 mg/kg BW) (21, 22). SPF grade male mice were fed a basal diet for 1 week. Thirty mice were randomly selected as diabetic model group according to body weight. After 4 weeks, all mice fasted for 12 h and were injected with STZ. Normal mice were intraperitoneally injected with the same amount of citrate buffer (pH = 4) for two consecutive times, and fasting blood glucose was measured 3 days later. Blood is collected through the caudal vein. After 1 week, the fasting blood glucose of mice exceeded 11.1 mmol/L for 7 days, and the mouse model of type 2 diabetes was established successfully.

#### 2.9.2. Grouping and dosing of animals

Mice were randomly divided into normal group (N), model group (M), positive control group (MET, metformin, 150 mg/kg), high-dose group (150 mg/kg), medium-dose group (100 mg/kg) and low-dose group (50 mg/kg). The positive control group and the low-dose, medium-dose and high-dose treatment groups were given metformin aqueous solution and germinated mung bean polyphenol aqueous solution, respectively, and the other groups were given constant volume of normal saline for continuous gavage for 5 weeks.

### 2.10. Determination of blood glucose related indexes in animals

Fasting for 12 h per week, fasting blood glucose (FBG) was measured. After the last administration, the mice fasted without water for 6 h, and were given 25% glucose solution at the dosage of 2 g/kg· BW to determine the blood glucose values at 0, 30, 60, 90, and 120 min. The area under the curve of blood glucose (AUC) was calculated according to formula, and the change of glucose tolerance (OGTT) was expressed by the area under the curve. During the experiment, the food intake, mental state and hair color of the animals were observed every day, and the weight of the mice was weighed on time every week. At the end of the experiment, the mice were fasted for 12 h, anesthetized with ether, took blood from the eyeballs, left for 30 min at room temperature, centrifuged at 3500 r/min for 10 min, took serum, and measured the content of insulin and islet β cells in serum (23, 24). After dissection, mouse liver, kidney, pancreas and other tissues were extracted, rinsed with normal saline, dried with filter paper, weighed, and the organ index was calculated according to the formula. The calculation formula involved is as follows:

$\begin{array}{l} \mathrm{AUC}\left(mmol/L\right)=\\ \;\;\;\;\;\;\;\;\;\;\;\;\;\;\;\;\;\;\;\;\;\;\;\;\frac{\left({\mathrm{FBG}}_{0}+{\mathrm{FBG}}_{30}\right)\times 0.5}{2}+\;\;\frac{\left({\mathrm{FBG}}_{30}+{\mathrm{FBG}}_{60}\right)\times 0.5}{2}+\\ \;\;\;\;\;\;\;\;\;\;\;\;\;\;\;\;\;\;\;\;\;\;\;\;\frac{F\left({\mathrm{BG}}_{60}+{\mathrm{FBG}}_{90}\right)\times 0.5}{2}+\frac{\left({\mathrm{FBG}}_{90}+{\mathrm{FBG}}_{120}\right)\times 0.5}{2}\\ \;\;\;\;\;\;\;\;\;\;\;\;\;\;\;\;\;\;\;\;\;\;\;\;Organ\;index\left(\%\right)=\frac{Organ\;weight\left(g\right)}{Total\;weight\left(g\right)}\times 100 \end{array}$


$$HOMA-IR=\frac{FBG\times FINS}{22.5}$$

Homeostasis model assessment-β (HOMA-β) = $\frac{20\times \mathrm{FINS}}{\mathrm{FBG}-3.5}$ .

### 2.11. Statistical analysis

Each experiment was repeated three times, and the data were expressed as the mean ± standard deviation. The significance was analyzed using IBM SPSS statistics 19.0 software, and the difference was significant if *p* < 0.05. The data were processed and plotted with Origin8.0 software.

## 3. Results

### 3.1. Effect of soaking and germination conditions on polyphenol content

**Figure 1:**
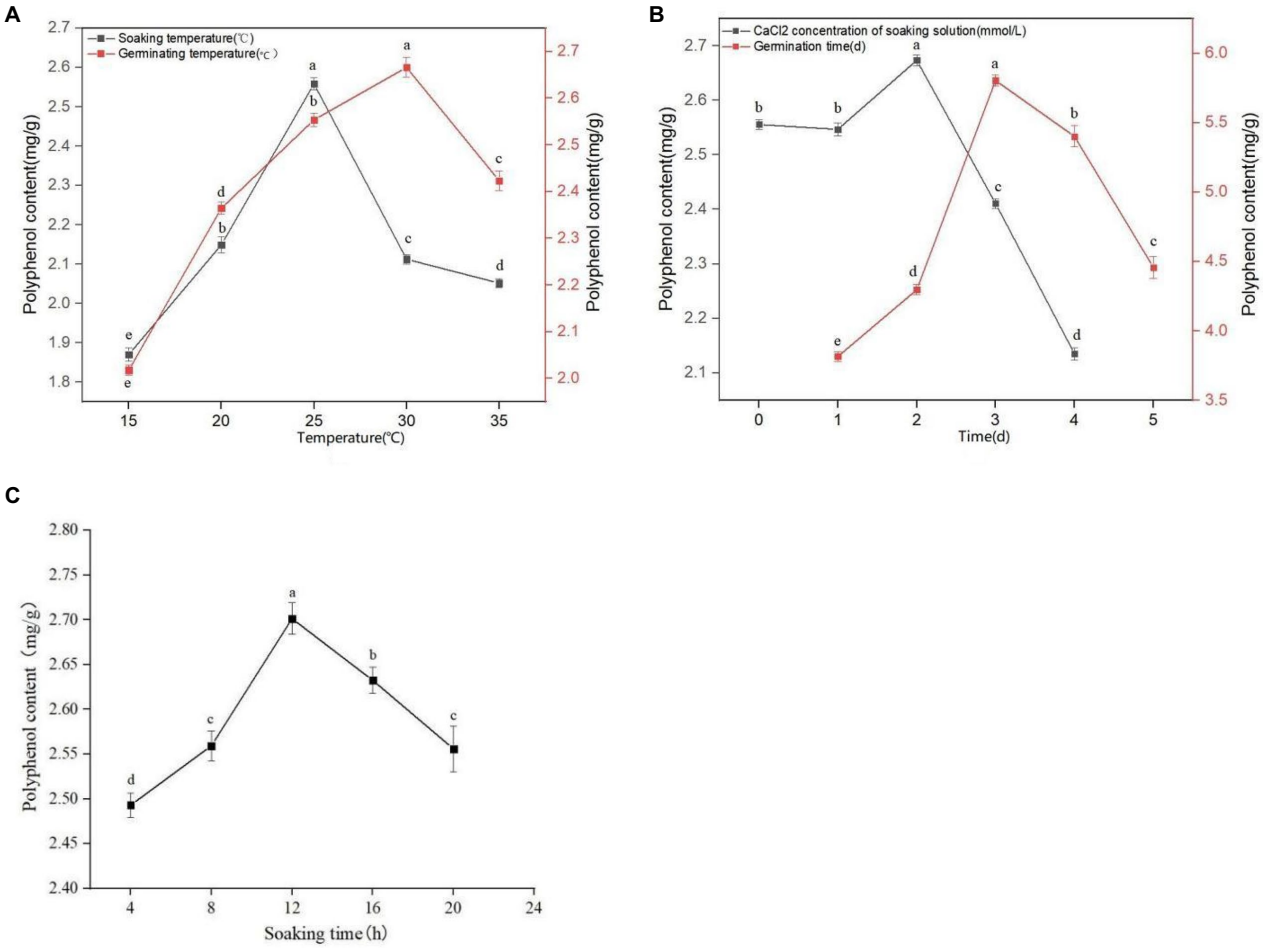
Effect of soaking temperature and germination temperature **(A)**, concentration of CaCl_2_ in soaking solution and germination time **(B)**, and soaking time **(C)** on polyphenol content of mung bean during germination. Data are mean ± standard deviation (*n* = 3). Different letters indicate significant differences.

It can be seen from Figure 1A that with an increasing soaking temperature, the quantity of extracted polyphenols from sprouted mung beans gradually increases, reaching a maximum value of 2.559 mg/g at a soaking temperature of 25°C. However, with a soaking temperature greater than 25°C, the concentration of polyphenols gradually decreases. The phenolic substances have poor thermal stability and are easily decompose on heating. Therefore, the optimal soaking temperature was selected as 25°C. It can be seen from Figure 1C that with increasing soaking time, the content of polyphenols in sprouted mung beans initially increases, and then decreases. At 12 h soaking time, the polyphenol extraction quantity reaches a maximum of 2.709 mg/g, whereas at a soaking time of 16 h, the phenolic components in the extraction solution decrease. With extensive soaking, the content of phenolic substances decreases because of the large number of phenolic hydroxyl groups with strong hydrophilicity. Therefore, some phenolic substances will be lost with longer soaking times, emphasizing the importance of determining the optimal soaking time, which we found to be 12 h. In the process of germination, the antioxidant activity of germinated mung bean can be improved by using trace element water. It can be seen from Figure 1B that germinated mung beans have the best adaptive concentration to CaCl_2_. A CaCl_2_ concentration of 2 mM, gives the optimal polyphenol extraction concentration. Increasing or decreasing the CaCl_2_ concentration reduces the polyphenol extraction quantity in germinated mung beans, with high concentrations affecting the extraction efficiency more than low concentration. With a CaCl_2_ concentration ≤ 1 mM, the germination of mung bean and the extraction quantity of polyphenols was not affected, whereas CaCl_2_ concentrations ≥3 mM significantly inhibited the polyphenol extraction from germinated mung beans. Therefore, the concentrations of 1, 2, and 3 mM were selected as the three levels of CaCl_2_ concentration in the immersion solution for response surface test design.

It can be seen from Figure 1A that the germination temperature has a great influence on the quantity of extracted polyphenols. At 30°C, the concentration of polyphenols reached a maximum of 2.684 mg/g. However, with higher germination temperatures, the extraction rate of polyphenols declined, likely due to thermal instability or volatile components being easily destroyed or volatilized. Therefore, the germination temperature of 30°Cwas selected for extraction. It can be seen from Figure 1B that the concentration of extracted polyphenols in germinated mung beans increase with increased germination time, reaching a maximum at 3 days germination. When germination time exceeds 3 days, the quantity of extracted polyphenol shows a downward trend. This may be due to the increased activities of various enzymes, as well as an increased period of respiration resulting in the oxidation or degradation of phenols. Moreover, the release and binding rates of phenols vary over time, which may result in a decrease in their content. Therefore, we choose the appropriate germination time of 3 days.

### 3.2. Response surface optimization experiment

#### 3.2.1. Response surface design and results

The Box–Behnken design was used to optimize the conditions for mung bean germination, and the a regression model was established to measure the effects of five independent variables, namely: soaking temperature (A), soaking time (B), germination temperature (C), germination time (D) and CaCl_2_ concentration (E), on polyphenol content are expressed. Design expert 8.0.6 was used to carry out quadratic multivariate regression fitting for the data, and the following quadratic multiple regression fitting equation was obtained: Y = 5.76 + 0.20×_1_ + 0.23×_2_ + 0.14×_3_ + 0.16×_4_-0.026×_5_ + 0.15X_1_X_2_–0.29X_1_X_3_–0.11X_1_X_4_ + 0.10X_1_X_5_–0.060X_2_X_3_–0.027X_2_X_4_–0.019X_2_X_5_ + 0.080X_3_X_4_ –0.31X_3_X_5_ + 0.051X_4_X_5_–1.70×_1_^2^-1.48×_2_^2^-0.94×_3_^2^-1.55×_4_^2^-1.52×_5_^2^.

The test results were analyzed by variance (Table 2): *F* = 31.09 and *p* < 0.0001 of the regression model of polyphenol content (Y) of germinated mung bean shows that the linear relationshipof the model is significant. The coefficient of determination (R^2^ = 0.9613) shows that this validationmethod is reliable and this model can be used to predict the optimize the extraction processof polyphenols from germinated mung beans. The order of influence of each factor on the concentration of polyphenols extracted from germinated mung beans is as follows: B (Soaking time) > A (Soaking temperature) > D (Germination time) > C (Germination temperature) > E (CaCl_2_ concentration).

**Table 2:** Analysis of variance in response surface regression model.

Source	Squares	df	Square	*F* value	Prob > F	Significance
Model	50.46	20	2.52	31.09	<0.0001	**
A (Soaking temperature)	0.61	1	0.61	7.54	0.0110	*
B (Soaking time)	0.87	1	0.87	10.66	0.0032	**
C (Germination temperature)	0.30	1	0.30	3.75	0.0640	
D (Germination time)	0.40	1	0.40	4.95	0.0354	*
E (CaCl_2_ concentration)	0.011	1	0.011	0.14	0.7149	
AB	0.093	1	0.093	1.15	0.2938	*
AC	0.34	1	0.34	4.16	0.0521	
AD	0.050	1	0.050	0.62	0.4381	
AE	0.043	1	0.043	0.53	0.4752	
BC	0.014	1	0.014	0.18	0.6784	
BD	2.809E-003	1	2.809E-003	0.035	0.8539	
BE	1.482E-003	1	1.482E-003	0.018	0.8936	
CD	0.026	1	0.026	0.32	0.5782	
CE	0.39	1	0.39	4.83	0.0375	*
DE	0.011	1	0.011	0.13	0.7220	
A^2^	25.24	1	25.24	311.01	<0.0001	**
B^2^	19.24	1	19.24	237.13	<0.0001	**
C^2^	7.67	1	7.67	94.48	<0.0001	**
D^2^	20.87	1	20.87	257.12	<0.0001	**
E^2^	20.29	1	20.29	250.02	<0.0001	**
Residual	2.03	25	0.081			
Lack of fit	1.80	20	0.090	1.92	0.2417	
Pure error	0.23	5	0.047			
cor total	52.49	45				
Coefficient of determination	0.9613					
Correction factor	0.9304					
Prediction coefficient	0.8568					

* * indicates extremely significant (*P* < 0.01), * indicates significant (0.01< *P* <0.05).

#### 3.2.2. Response surface interaction analysis

**Figure 2:**
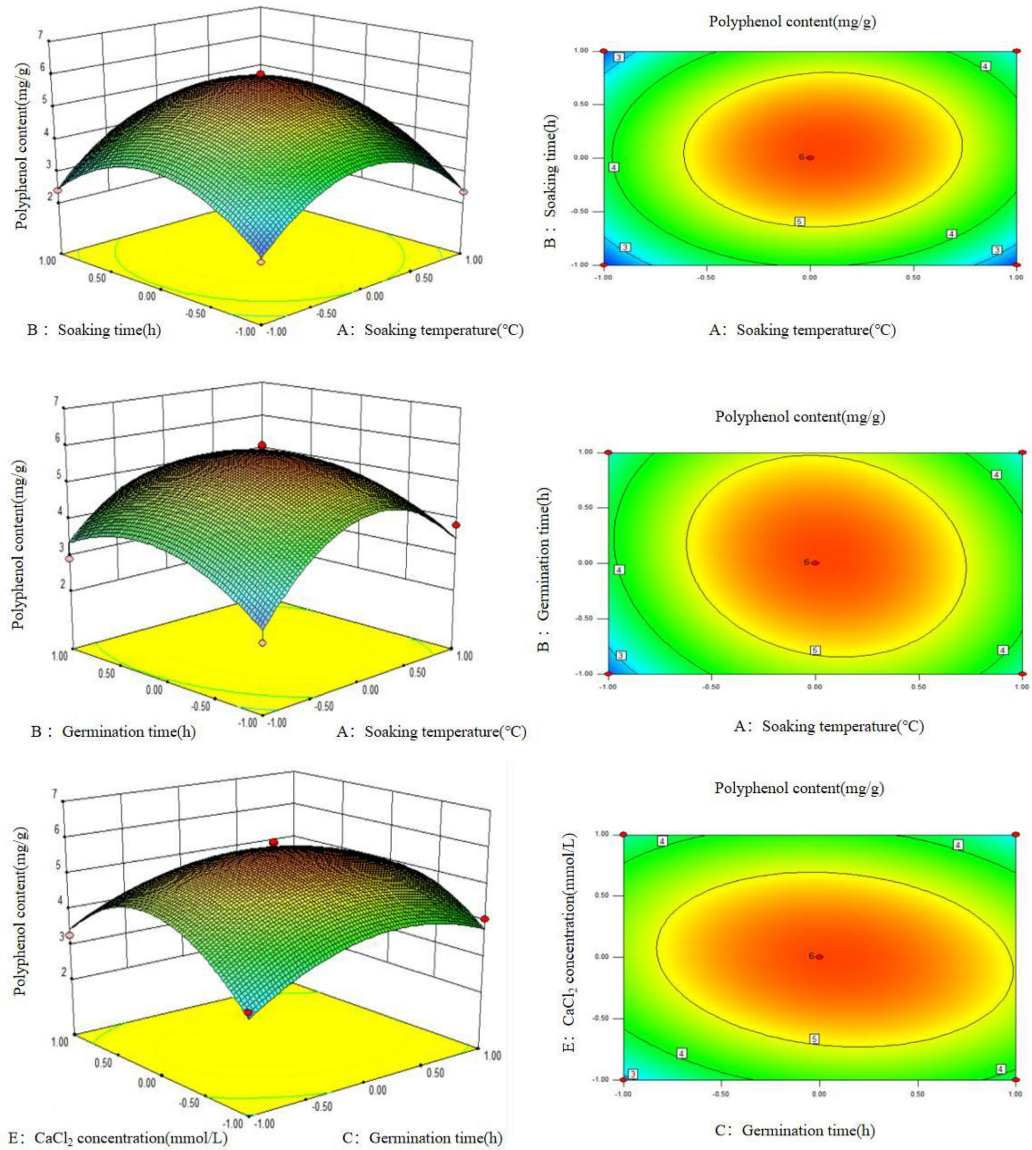
Contour lines and response surface diagrams of the interaction between AB, AC, and CE.

It can be seen from the response surface graph that the surface for soaking time, soaking temperature, and germination time is curved, indicating that these three factors have a significant effect on the quantity of polyphenols extracted from germinated mung beans (Figure 2). In addition, the contour map shows that the soaking temperature, soaking time, germination time, and CaCl_2_ concentration have a significant impact on the extraction quantity of polyphenols extracted from germinated mung beans, while the interaction of other factors is not significant, which is consistent with the ANOVA result.

Using design expert 8.0.6 software to optimize the parameters of polyphenol extraction from germinated mung bean, we found an optimal polyphenol extraction quantity of 5.01 mg/g. The most optimal extraction conditions were as follows: a soaking temperature of 25°C, soaking time of 11 h, germination temperature of 28°C, germination time of 3 days, CaCl_2_ concentration of 2 mM. Through three parallel experiments, we measured the polyphenol content of mung bean after germination was (4.878 ± 0.30) mg/g, which was 3.07 times higher than that of mung bean without germination.

### 3.3. Isolation and purification of polyphenols from germinated mung bean

**Figure 3:**
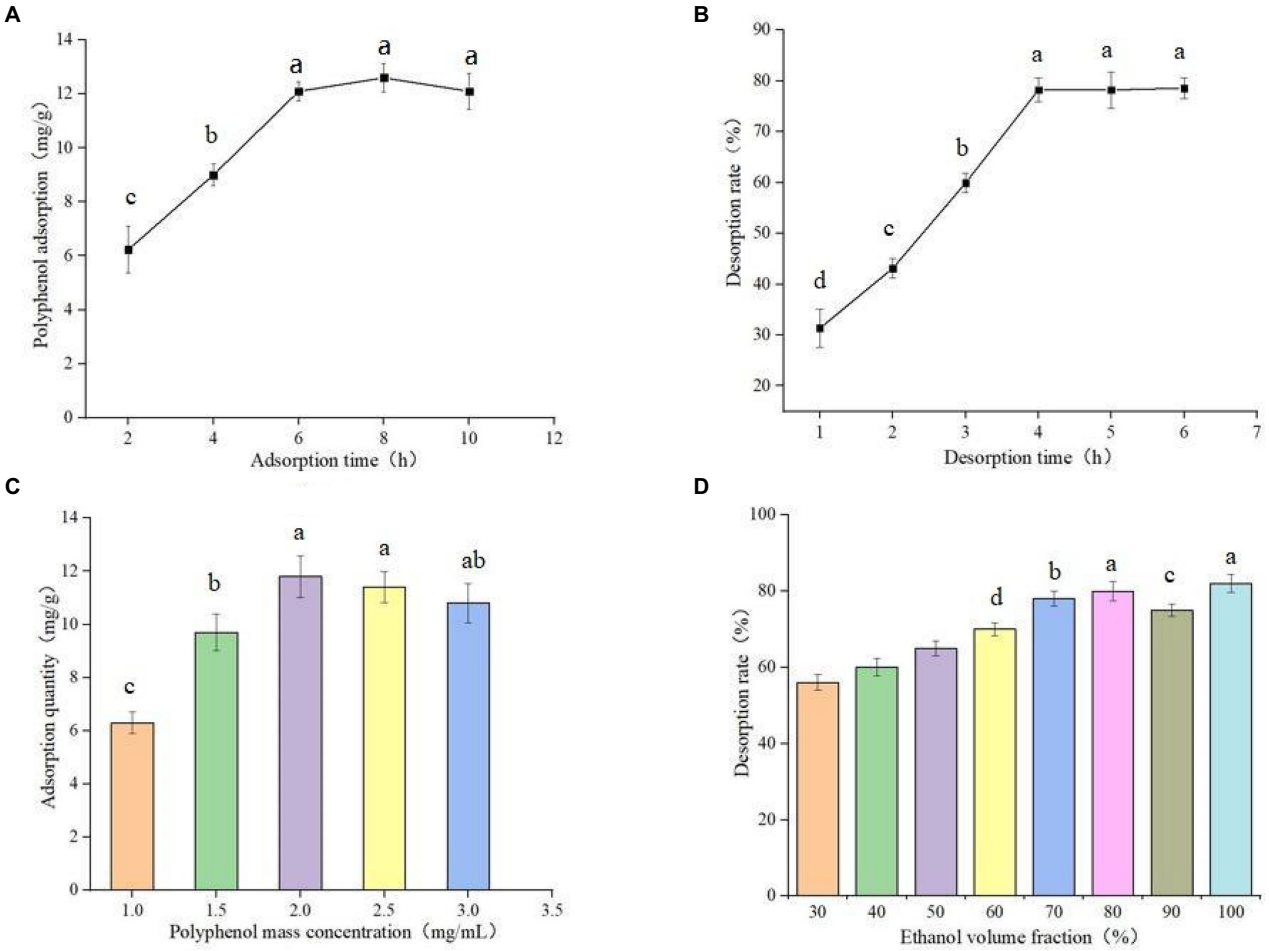
Static adsorption curve of AB-8 macroporous resin **(A)**, Desorption curve of AB-8 macroporous resin **(B)**, Effect of different polyphenol concentration on adsorption capacity of germinated mung bean **(C)** and Effect of ethanol volume fraction on desorption rate **(D)**. Data are mean ± standard deviation (*n* = 3). Different letters indicate significant differences.

The experimental results of static adsorption and static desorption are shown in Figure 3A. The adsorption time was 8 h, and the adsorption amount was 12.81 ± 0.78 mg/g. The fast adsorption speed before 6 h may be due to the increase of adsorption surface area after the macroporous resin was soaked completely, whereas the slow adsorption speed after 6 h may be due to the gradual saturation of adsorption. As shown in Figure 3B, the desorption time reached full saturation at 4 h, and the desorption rate was 79.83 ± 0.96%. Therefore, the static adsorption and desorption times were determined to be 8 and 4 h, respectively.

The experimental results of different polyphenol mass concentrations on the adsorption capacity are shown in Figure 3C. When the polyphenol mass concentration is between 1.0–2.5 mg/ml, the adsorption capacity gradually increased polyphenol mass concentration. When the polyphenol mass concentration is between 2.0–2.5 mg/ml, the adsorption capacity does not change significantly, and when the adsorption capacity increases, the adsorption capacity decreases. When the mass concentration of the sample is too high, this blocks the macroporous resin and reduces the adsorption capacity of the resin. Therefore, 2.0 mg/ml was selected as the optimal polyphenol mass concentration of the sample. The effect of different concentrations of eluates on the content of polyphenols is shown in Figure 3D. The concentrations of eluates correlates with the desorption rates, and it gradually increases with increased ethanol volume fraction. When the concentration of ethanol is more than 80%, the change of desorption rate tends to be gentle. However, there is no significant difference between using 80% ethanol solution and absolute ethanol as the eluent: therefore we chose 80% ethanol solution as the eluate concentration.

### 3.4. Structure identification of polyphenols in germinated mung bean

**Figure 4:**
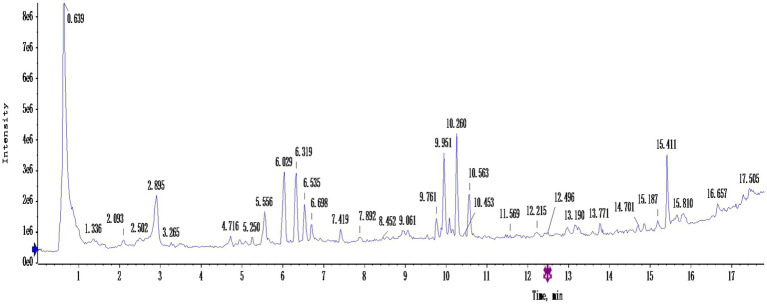
Total ionogram of purified germinated mung bean polyphenols.

Figure 4 shows the total ion flow diagram of germinated mung bean polyphenols after purification, and Table 3 shows the identification of main components of germinated mung bean polyphenols. By using HPLC-MS/MS to determine the total ion flow pattern of germinated mung bean polyphenols and the mass/charge ratio and molecular mass of the compounds, and by referring to the database and existing studies, we can preliminatively conclude that the main compounds contained in germinated mung bean polyphenols are: Quinic acid, Quercetin, Rutin, Vitexin, Isovitexin, etc. The content of polyphenols in germinated mung bean was 65.19%.

**Table 3:** Identification of main components of polyphenols in germinated mung bean.

Peak	t_R_ (min)	Molecular (M-)	Fragment (m/z)	Formula	Identification	CAS No.
1	0.65	191	59,87,127	C_7_H_12_O_6_	Quinic acid	77–95-2
2	2.895	301	85,151,273	C_15_H_10_O_7_	Quercetin	117–39-5
3	5.563	609	254,300	C_27_H_30_O_16_	Rutin	153–18-4
4	6.319	431	283,341	C_21_H_20_O_10_	Vitexin	3,681-93-4
5	6.533	431	341	C_21_H_20_O_10_	Isovitexin	38,953–85-4
6	9.761	267	211	C_15_H_8_O_5_	Coumestrol	207–525-6
7	10.08	353	135,191	C_16_H_18_O_9_	Chlorogenic acid	327–97-9
8	10.260	293	236,292	C_17_H_26_O_4_	Embelin	550–24-3
9	10.56	941	941,903	C48H78O18	Soyasaponin Bb	51,330–27-9
10	15.41	339	338,341	C_20_H_20_O_5_	8-Prenylnaringenin	53,846–50-7

### 3.5. Inhibitory activity of polyphenols from germinated mung bean on α-glucosidase *in vitro*

**Figure 5:**
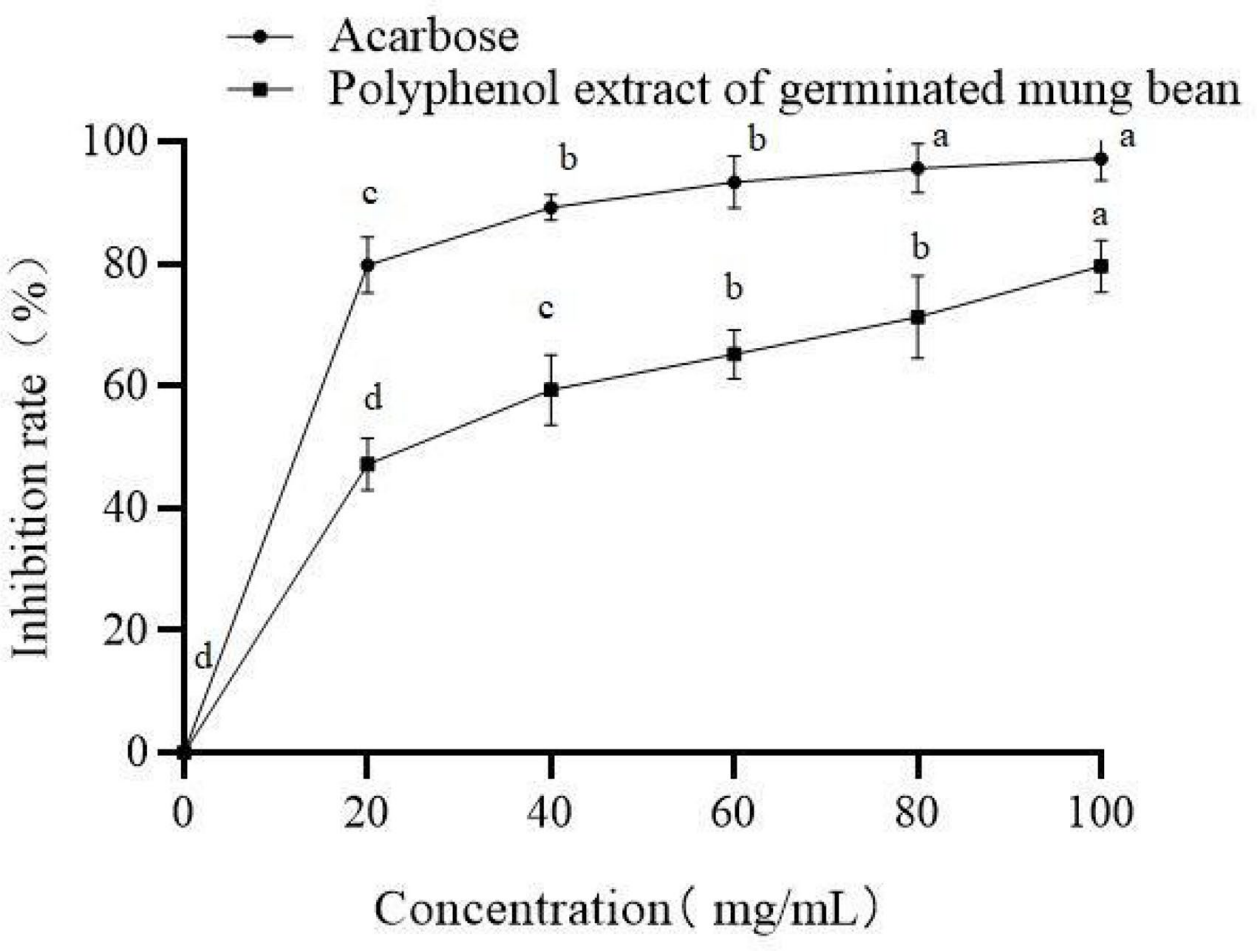
Inhibitory activity of Germinated mung bean polyphenols and acarbose on α-glucosidase. Data are mean ± standard deviation (*n* = 3). Different letters indicate significant differences.

**Figure 6:**
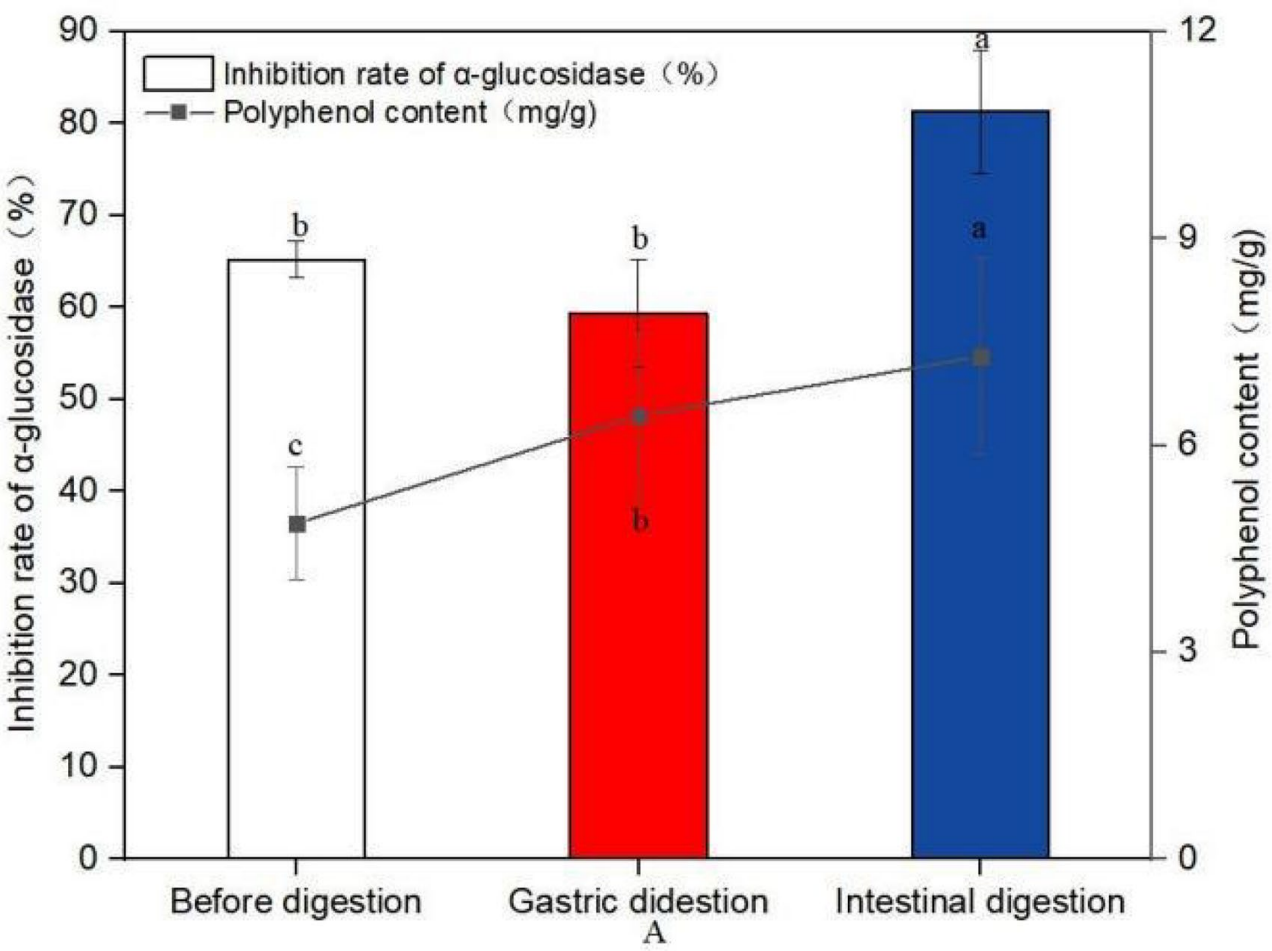
Changes of polyphenol content and inhibition rate of glucosidase before and after digestion (A). Data are mean ± standard deviation (*n* = 3). Different letters indicate significant differences.

Eating foods that inhibit α-glucosidase activity can effectively alleviate the rise of postprandial blood glucose concentration, which is conducive to the prevention and control of diabetes. As shown in Figure 5, germinated mung bean polyphenols have an obvious dose-dependent inhibitory effect on α-glucosidase activity. When the mass concentration of polyphenols was 100 mg/ml, the inhibition rate of α-glucosidase was as high as 79.6%. Acarbose was selected as the positive control in this experiment, with an IC_50_ of 16.32 mg/ mL. The IC_50_ of germinated mung bean polyphenols was 44.45 mg/ml. This demonstrates that germinated mung bean polyphenols had an obvious inhibitory effect on α-glucosidase, and the inhibitory activity showed a dose-dependent relationship (Figure 6).

### 3.6. Effects of *in vitro* digestion on polyphenol content and hypoglycemic activity *in vitro*

The polyphenol content of sprouted mung beans before digestion was 4.87 mg/g, and after digestion of gastric juice, the polyphenol content increased to 6.43 mg/g, which was 32.03% higher than that of undigested mung beans. After intestinal digestion, the content of polyphenols was 7.29 mg/g, which was 49.69% higher than that of undigested polyphenols. It shows that *in vitro* digestion is more conducive to the increase of polyphenol content.

Compared with the polyphenol like solution before digestion, the inhibitory rate of the polyphenol after gastrointestinal digestion on α-glucosidase increased by 24.69%. This experiment proved that the sprouted mung bean polyphenols still had good hypoglycemic activity *in vitro* after simulated digestion *in vitro*.

### 3.7. Effect of polyphenols on type 2 diabetic mice

#### 3.7.1. Effects of polyphenols on the results of basal indexes in mice

**Figure 7:**
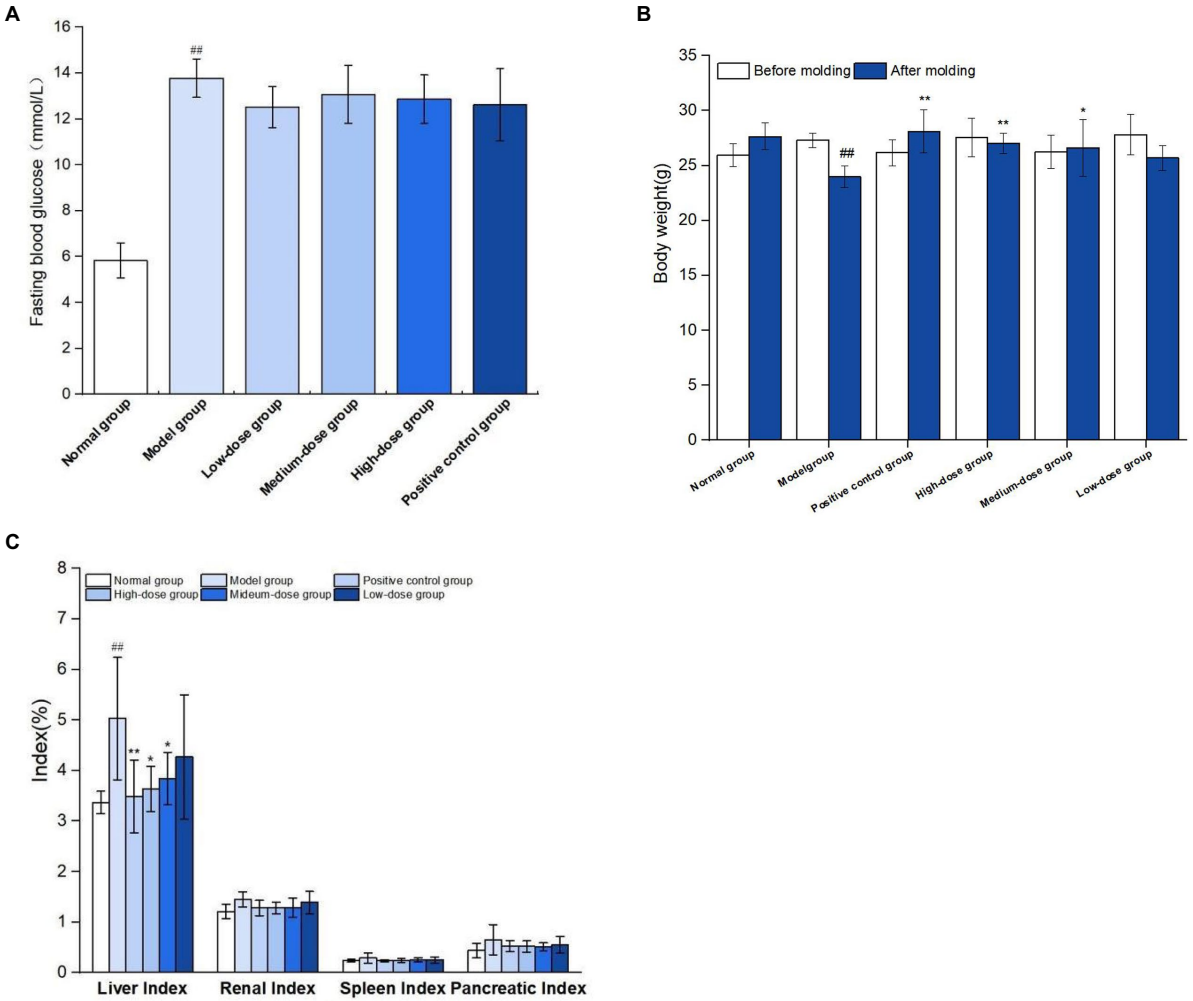
Results of T2DM modeling index **(A)**, effect of germinated mung bean polyphenols on body weight of mice in each group **(B)**, and effect of germinated mung bean polyphenols on organ index in diabetic mice **(C)**. The data in the figure are represented as mean ± SD (*n* = 6). Difference significance:* means *p* < 0.05, ** means *p* < 0.01, compared with the model group, and ## means *p* < 0.01, compared with the normal group.

As shown in Figure 7A, on the 7th day after modeling, fasting blood glucose levels of mice were all over 11.1 mmol/L, indicating that the model of mice with type 2 diabetes was successfully constructed. During modeling, normal group was fed basic diet, model group and germinated mung bean polyphenol dose group were fed high-fat diet, and type 2 diabetes mouse model was induced by STZ combined with STZ at the 4th week of feeding. After the modeling, it was found that the mental state of the mice in the normal group was good, the hair was smooth, the food intake and water intake were relatively stable before and after the experiment, and the body weight gradually increased. The spirit of the diabetic model group was weak, and the hair was dull and lustrous. Compared with before the experiment, the mice showed “three and more” symptoms. The weight loss of mice in positive control group and each dose group slowed down. As shown in Figure 7B, after successful modeling, the body weight of mice in the model group decreased significantly compared with that in the normal group (P<0.01). At the end of the experiment, compared with the model group, the body weight of mice in the medium-dose group of germinated mung bean polyphenol solution was significantly increased (P<0.05), and the body weight of mice in the high-dose group and the metformin positive control group was significantly increased (P<0.01), by 10.80 and 12.50%, respectively.

As shown in Figure 7C, the liver index of mice in the model group was significantly increased by 49.26% compared with the normal group. Liver indexes in both the medium-dose and high-dose groups were significantly lower than in the model group. The results showed that the liver swelling and kidney swelling were obvious in T2DM mice, and the germinated mung bean polyphenols could reduce the liver swelling of T2DM mice after intervention, indicating that both the positive drug group and germinated mung bean polyphenols could improve the organ index of type 2 diabetes mice, and the polyphenol extract has the characteristics of natural safety and non-toxic side effects.

#### 3.7.2. Effects of germinated mung bean polyphenols on blood sugar related indexes in type 2 diabetic mice

**Figure 8:**
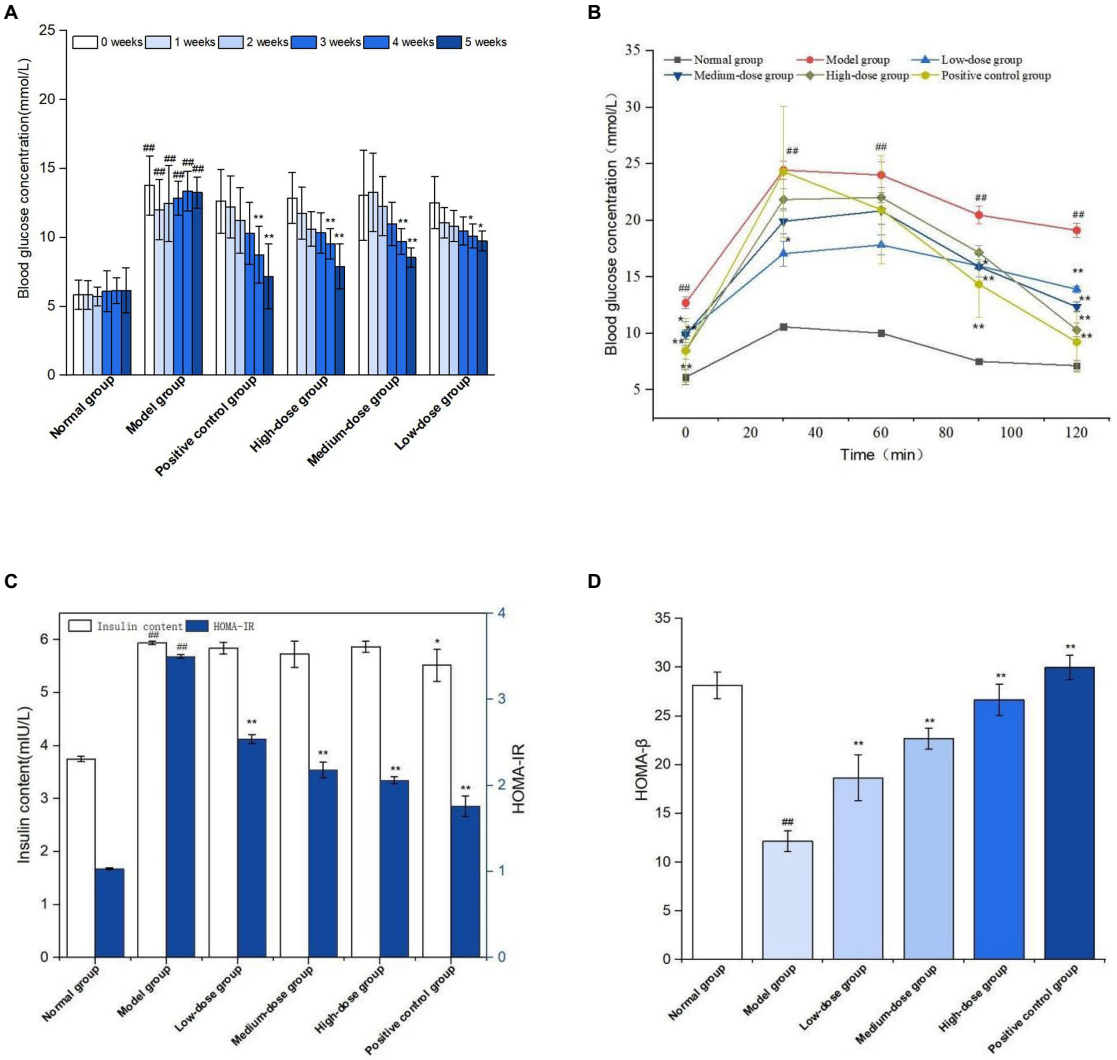
Fasting blood glucose levels during GMP intervention **(A)** oral glucose tolerance **(B)** Fasting insulin and insulin resistance index **(C)** Islet β cell function index **(D)**. The data in the figure are represented as mean ± SD (*n* = 6). Difference significance:* means *p* < 0.05, ** means *p* < 0.01, compared with the model group, and ## means *p* < 0.01, compared with the normal group.

The effect of sprouted mung bean polyphenols on the blood sugar of diabetic mice is shown in Figure 8A. The fasting blood glucose concentration of mice in the normal group is relatively stable, while that of mice in the model group gradually increases after the establishment of the diabetic model. At the end of the experiment, the fasting blood glucose concentration of the germinated mung bean polyphenol group was significantly lower than that of the model group, and the fasting blood glucose values of the medium-dose and high-dose groups were significantly reduced. Fasting blood glucose measurements showed that germinated mung bean polyphenols could improve the blood glucose level of type 2 diabetic mice during the intervention period.

Glucose tolerance experiment is shown in Figure 8B. The blood glucose concentration of mice in the normal group tends to change gently over time, while the blood glucose level of mice in the model group is at a relatively high level although it shows a downward trend. The metformin positive group and germinated mung bean polyphenol high dose group showed a significant decrease in blood glucose concentration, but did not return to the initial blood glucose value. According to the glucose tolerance test, the areas under the curve of each group were calculated as follows: 18.02 in the normal group, 41.96 in the model group, 34.49 in the positive control group, 31.41, 33.90, and 35.21 in the germinated mung bean polyphenols low-dose, medium-dose and high-dose groups, respectively. The areas under the curve decreased more significantly in the high-dose group and the positive control group.

Insulin is very important for the regulation of blood sugar. As shown in Figure 8C, FINS level and HOMA-IR index of mice in the diabetic model group were significantly increased compared with the normal group, while insulin levels in the positive control group and germinated mung bean polyphenol low-dose, medium-dose and high-dose groups were decreased without significant difference, but HOMA-IR index levels were significantly decreased. HOMA-β is an important indicator of islet beta cell function, which decreases when diabetes is severe. As shown in Figure 8D, compared with the model group, HOMA-β index in the positive control group and the germinated mung bean polyphenol group was significantly increased, with the high dose group having the most significant effect. The results showed that germinated mung bean polyphenol extract can increase insulin sensitivity, reduce insulin resistance and repair the function of pancreatic beta cells in type 2 diabetic mice.

## 4. Discussion

Mung bean is a leguminous crop rich in bioactive substances, which many studies have shown to have the potential to promote health and prevent disease (11). Studies have shown that germinated mung beans increase their nutritional value and enhance their health-promoting benefits (25). Germination treatment can significantly reduce or transform non-nutritive components, thus improving nutrient utilization (26). Germination can activate the respiratory system of pulses, produce different types of endogenous enzymes, hydrolyze starch, protein, and degrade the cell wall. During the process, the content of trace elements and the level of active components change. For example, the content of protein and mineral, flavonoids, total phenols and free phenols, and antioxidant capacity increase after germination (27). However, the increase of phenolic substances is not necessarily proportional to the germination time but is also affected by other factors. For example, soaking treatment can dissolve the free polyphenols, and also destroy the stable structure of some polyphenols in beans and other substances, further leading to the outflow of bound polyphenols, and ultimately increasing the content of polyphenols. Bishnoi et al. (28) showed that the content of total phenolic compounds in brown rice increased during 24 h immersion; Yang et al. (29) found that the content of phenols in wheat increased after soaking. Sharma S et al. showed that millet soaked at room temperature for 15.84 h and germinated at 25°Cfor 40 h, its total phenol content was significantly increased (30). Kim DK et al. showed that the phenolic substances, total flavone content and DPPH radical scavenging activity of the germinated extract were higher than those of the non-germinated seed extract (31). In this experiment, the content of polyphenols was increased by soaking treatment, but the results showed that the content of polyphenols first increased and then decreased with soaking time, which was consistent with the above experimental results. This study also investigated the effect of CaCl_2_ concentration in soaking solution on the content of polyphenols in germinated mung bean. The experimental results showed that the appropriate concentration of CaCl_2_ could promote the germination of mung bean and increase the content of polyphenols. Calcium is a structural element of plant cells. During the germination of mung bean, Ca^2+^ can activate a variety of enzymes in the body and improve the metabolic rate of substances. It plays an important role in the germination process and can improve the germination rate of mung bean. Studies have shown that appropriate concentration of Ca^2+^ can promote the growth of sprouts, shorten the production cycle of sprouts, improve nutritional quality, low concentration of Ca^2+^ can improve the germination rate of mung bean, promote the state of sprouts, but too high Ca^2+^ will damage mung bean, has an inhibitory effect on the germination of mung bean. The influence of temperature on germination is also crucial, because polyphenols undergo monomer transformation during the process of temperature rise, and the degradation rate of phenols and the outflow rate of bound phenols may be different among different varieties of beans. Therefore, in this experiment we aimed to optimize the germination conditions of mung bean, to maximize the quantity of phenolic substances released. The results showed that the polyphenol content of germinated mung bean was 3.07-times higher than that of non-germinated mung bean under the optimal germination conditions. It shows that the content of phenols in mung beans will increase significantly after germination, which is consistent with the conclusions of other people’s experiments above.

Related literature reported that the increase of polyphenol content can enhance the hypoglycemic activity. However, hypoglycemic activity depends not only on the content of polyphenols, but may also depend on the composition of polyphenols. Therefore, phenolic structures play an important role in biological activity (32). According to the report, not sprout mung bean sprout and 3 days of mung bean samples contrast, there are several common phenolic compounds, such as genistein, kaempferol-3-O-rutinase, quercetin, rutin and vitexin. However, new polyphenol compounds may be induced during germination. In this experiment, in order to further determine the structural type and content of phenolic acids that play an hypoglycemic role, HPLC-MS/MS method was used to quantitatively analyze the structure and content of main phenolic substances. Many rich polyphenols were identified, including Quinic acid, Quercetin, Rutin, Vitexin, Isovitexin, etc. Through structural analysis and combining with other research literature, it is speculated that the hypoglycemic activity of polyphenols in germinated mung bean may be highly correlated with the structure and content of Quinic acid, Quercetin, Rutin, Vitexin, Isovitexin and other substances. The results showed that the content of polyphenols in mung beans not only increased after germination, but also changed the specific components of phenols, indicating that the hypoglycemic activity of polyphenols was related to the content and components of phenols. Or some components of phenols will play a synergistic role in hypoglycemic activity. Different processing methods will affect the phenolic substances and enzyme inhibition characteristics in plants. We investigated the inhibitory effect of germinated mung bean polyphenol extract on α-glucosidase. The experimental results show that compared with non-sprouted mung beans, sprouted mung beans have higher polyphenols and higher inhibition activity to enzymes, with an IC_50_ of 44.45 mg/ml, and the inhibitory activity showed a dose-dependent relationship with the mass concentration of polyphenols. The content and structure of polyphenols are important factors affecting antioxidant capacity and α-glucosidase activity. Similar to the experimental results of Ameenah and Messina (33, 34). Randhir et al. (35) found that the inhibition potential of the bud extract on α-amylase was relatively high in the early stage (0–2 days) and significantly increased in the 4–10 days, which was related to the higher phenol content. Qin et al. (36) found that soaking tartary buckwheat significantly increased the contents of quercetin, kaempferol, total flavonoids and total phenolic compounds, and these components had a significant impact on the inhibitory activity of α-glucosidase. *In vitro* simulated digestion model is often used to study a method to simulate the digestion and absorption process of bioactive substances in human body under *in vitro* experimental conditions. The digestion and absorption process of food *in vivo* is relatively complex, for example, the content and composition of polyphenols will change greatly during digestion, and may even directly affect their biological activity. Therefore, through the simulated gastrointestinal digestion experiment, this experiment proved that the content of polyphenols will increase after digestion, and has good hypoglycemic activity *in vitro*. The possible reason is that the form of polyphenols in the body has changed after they interact with digestive enzymes. Through the process of digestion in the body, they combine with proteins, lipids and other biological macromolecules to form other complexes, which further effectively improve their hypoglycemic activity. However, the specific mechanism of action needs further in-depth study.

In order to further determine the hypoglycemic activity of polyphenol extract from germinated mung bean, α-glucosidase activity was determined. In this study, high-fat diet combined with STZ was used to induce type 2 diabetes mice model to demonstrate the hypoglycemic activity of germinated mung bean polyphenol extract. The experiment showed that a certain dose of germinated mung bean polyphenols can improve the “three more and one less” symptoms of T2DM mice, reduce the blood glucose concentration of diabetic mice during the intervention period, and enhance the degree of sugar tolerance and insulin resistance to a certain extent. In this study, it was found that the germinated mung bean polyphenol extract dose group significantly reduced the organ index after intervention, which may improve the organ function of type 2 diabetes mice. In recent years, many studies have shown that a diet rich in polyphenols is effective in preventing and managing T2DM. Polyphenols extracted from legumes (37), can inhibit glucose transport, and eating a diet rich in polyphenols can greatly reduce the risk of diabetes (38), possibly because polyphenols are beneficial to glucose metabolism and can significantly improve glucose tolerance (39). The results of this study indicate that the function of germinated mung bean polyphenol extract in regulating blood glucose has been confirmed, but the mechanism of lowering blood glucose needs further study.

## 5. Conclusion

In this study, the process of mung bean sprouting and polyphenol enrichment was optimized by response surface methodology, and the optimal process parameters were determined as: soaking temperature 25°C, soaking time 11 h, germination temperature 28°C, germination time 3d, and CaCl_2_ concentration 2 mM. Under these conditions, the polyphenol content was 4.87 mg/g, which was 3.07 times higher than that of non-germinated mung bean. HPLC-MS/MS analysis showed that the main substances identified in the polyphenol samples of sprouting mung bean after purification by AB-8 macroporous adsorption resin included Quinic acid, Quercetin, Rutin, Vitexin, Isovitexin, etc. According to the detection results, the content of purified germinated mung bean polyphenols was 65.19%. This shows that the content of polyphenols can be effectively increased by purification. In addition, germinated mung bean polyphenols showed dose-dependent inhibition of α-glucosidase and continued to show inhibition after digestion *in vitro*, indicating that it has hypoglycemic activity *in vitro*. Our experiment also confirmed that germinated mung bean polyphenol extract can reduce fasting blood glucose, improve glucose tolerance and reduce insulin resistance in diabetic C57BL/6 mice, and has a protective effect on type 2 diabetic mice. In summary, it is proved that sprouted mung bean polyphenols, as a natural hypoglycemic substance with certain development potential and research value, have a wide prospect in the future application of food and medicine.

## Data availability statement

The original contributions presented in the study are included in the article/Supplementary material, further inquiries can be directed to the corresponding author.

## Ethics statement

The animal study was reviewed and approved by Animal Care Committee, College of Animal Science and Veterinary Medicine, Heilongjiang Bayi Agricultural University.

## Author contributions

BL was responsible for the design of the whole experiment and wrote the manuscript. XS helped the instrument carry out some experiments. HS collected and sorted out the experimental data. YZ helped analyze the data. XY helped revise the manuscript. All authors contributed to the article and approved the submitted version.

## Funding

This work was supported by the National technical system of rice industry (CARS-01-50) and Scientific research funds of Heilongjiang provincial Support from research institutes (CZKYF2023-1-B030).

## Conflict of interest

The authors declare that the research was conducted in the absence of any commercial or financial relationships that could be construed as a potential conflict of interest.

## Publisher’s note

All claims expressed in this article are solely those of the authors and do not necessarily represent those of their affiliated organizations, or those of the publisher, the editors and the reviewers. Any product that may be evaluated in this article, or claim that may be made by its manufacturer, is not guaranteed or endorsed by the publisher.
